# External validation of the de novo stress urinary incontinence prediction model after pelvic organ prolapse surgery in Korean women: a retrospective cohort study

**DOI:** 10.1186/s12905-023-02812-3

**Published:** 2023-12-08

**Authors:** Min Ju Kim, Youjoung Lee, Dong Hoon Suh, Sungyoung Lee, Myung Jae Jeon

**Affiliations:** 1 Department of Obstetrics and Gynecology, Seoul National University Hospital, Seoul, Korea; 2 Department of Obstetrics and Gynecology, Seoul National University Bundang Hospital, Seongnam, Korea; 3 Department of Genomic Medicine, Center for Precision Medicine, Seoul National University Hospital, Seoul, Korea; 4 Department of Obstetrics and Gynecology, Seoul National University College of Medicine, 103 Daehak-ro, Jongno-gu, Seoul, 03080 Korea

**Keywords:** External validation, Pelvic organ prolapse, Prediction model, Stress urinary incontinence

## Abstract

**Background:**

De novo stress urinary incontinence (SUI) may develop following pelvic organ prolapse surgery. Performing prophylactic continence surgery may reduce the risk of de novo SUI and subsequent continence surgery; however, it may increase the risk of complications. Therefore, many surgeons try to identify women at high risk for de novo SUI and perform continence surgery selectively. Recently, a model for predicting the risk of de novo SUI after prolapse surgery was developed using data from the Outcomes following vaginal Prolapse repair and midUrethral Sling (OPUS) trial; its prediction accuracy was significantly better than that of the stress test alone. However, few studies have verified its prediction accuracy in discrete populations. The aim of this study was to externally validate the prediction model for de novo SUI after prolapse surgery in Korean women.

**Methods:**

This retrospective cohort study included 320 stress-continent women who underwent prolapse surgery for pelvic organ prolapse quantification stage 2–4 anterior or apical prolapse and who completed a 1-year follow-up. Predicted probabilities by the de novo SUI online risk calculator were compared with observed outcomes and quantitated using the model’s area under the curve and calibration plot. Subgroup analyses were also performed by the type of prolapse surgery.

**Results:**

The de novo SUI prediction model showed moderate discrimination in our study cohort; area under the curve (95% confidence interval) = 0.73 (0.67–0.78) in the whole cohort, 0.69 (0.61–0.78) in women who underwent native tissue repair or colpocleisis, and 0.74 (0.65–0.82) in those who underwent sacrocolpopexy. Calibration curves demonstrated that the model accurately predicted the observed outcomes of de novo SUI in women who underwent native tissue repair or colpocleisis but underestimated outcomes in those who underwent sacrocolpopexy. The predicted probability cutoff points corresponding to an actual risk of 50% were 40% in women who underwent native tissue repair or colpocleisis and 30% in those who underwent sacrocolpopexy.

**Conclusions:**

The de novo SUI prediction model is acceptable for use in Korean women and may aid in shared decision-making regarding prophylactic continence procedure at the time of prolapse surgery.

## Background

De novo stress urinary incontinence (SUI) may develop after surgical correction of pelvic organ prolapse (POP) in stress-continent women and this is believed to be due to a correction of anatomical urethral kinking or compression from advanced prolapse. Performing prophylactic continence surgery may reduce the risk of de novo SUI and subsequent continence surgery; however, it may increase the risk of complications, such as bladder or urethral injury, tape exposure and voiding difficulty [1, 2]. For this reason, many surgeons try to identify women at high risk for de novo SUI and perform continence surgery selectively.

One common strategy uses a preoperative prolapse reduction stress test. If occult SUI is detected, a continence procedure is performed at the time of prolapse surgery. If occult SUI is not detected, prolapse surgery alone is performed [3]. However, the preoperative prolapse reduction test does not accurately predict postoperative SUI; 40% of women who do not undergo continence surgery with negative testing will develop postoperative SUI [4]. Recently, a model for predicting the risk of de novo SUI after prolapse surgery was developed using data from the Outcomes Following Vaginal Prolapse Repair and Midurethral Sling (OPUS) trial; its prediction accuracy was significantly better than that of the stress test alone [5]. However, few studies have verified its prediction accuracy in discrete populations [6–8].

The aim of this study was to externally validate the prediction model for de novo SUI after prolapse surgery in Korean women.

## Methods

After obtaining approval from the institutional review board for this retrospective cohort study (Seoul National University College of Medicine/Seoul National University Hospital 1908-018-1053), we reviewed the medical records of 583 stress-continent women who underwent surgery for pelvic organ prolapse quantification stage 2–4 anterior or apical prolapse at two tertiary hospitals in South Korea (Seoul National University Hospital and Seoul National University Bundang Hospital) between October 2008 and September 2018. Stress-continent was determined by as a negative response to question 17 (urine leakage related to coughing, sneezing or laughing) on the Korean version of the Pelvic Floor Distress Inventory Short Form [PFDI-20] questionnaire [9]. Among them, 263 women, who had a history of prolapse or anti-incontinence surgery, did not undergo prolapse reduction stress test or did not complete a 1-year follow-up, were excluded from the analysis; the data of 320 women were analyzed.

Data on seven parameters of the prediction model, including age at surgery, body mass index, vaginal parity, diagnosis of diabetes mellitus, preoperative urgency urinary incontinence (defined as an affirmative response to question 16 on the PFDI-20) [9], preoperative prolapse reduction stress test result and concomitant continence procedure, were collected from the medical records. The predicted probability of de novo SUI was calculated using an online risk calculator (https://riskcalc.org/FemalePelvicMedicineandReconstructiveSurgery) [10].

All patients were followed up postoperatively at 1, 4–6, and 12 months and then annually thereafter. De novo SUI was defined as the presence of bothersome SUI symptoms at the 4–6 or 12-month visit, determined by a response of ‘somewhat’, ‘moderately’ or ‘quite a bit’ bothersome to question 17 on the PFDI-20 and/or subsequent continence procedure within 12 months postoperatively.

All statistical analyses were performed using R statistical software and its associated packages. Model discrimination was quantified using the area under the receiver operating characteristic curve (AUC); a value of 1 indicated perfect discrimination between those who experienced de novo SUI and those who did not, 0.75 was considered good discrimination, 0.51 to 0.74 was considered moderate and 0.5 indicated no better than chance [11]. Model calibration was assessed by visual inspection of the calibration plot. Calibration curves visualize how accurate the predicted probability of the model is to the observed outcome, where a perfect correlation followed a straight 45-degree line. Subgroup analyses were also performed to determine whether the model’s performance varied for the type of prolapse surgery.

## Results

Baseline demographic and clinical data of the study population are summarized in Table 1. The median age, body mass index and vaginal parity were 68.0 (63.0–73.0) years, 24.7 (22.8–26.7) kg/m^2^ and 3 (2–4), respectively. Fifty-five (17.2%) women reported a history of diabetes mellitus, and 115 (35.9%) had preoperative urgency urinary incontinence. One hundred and sixty-eight (52.5%) women had a positive preoperative prolapse reduction stress test result, and concomitant midurethral sling (MUS) was performed on 148 (46.3%) women with a positive stress test result in the manner of transobturator tape.

**Table 1:** Characteristics of the study population (n = 320)

Variable	Value
Age at surgery, yr	68.0 (63.0–73.0)
Body mass index, kg/m^2^	24.7 (22.8–26.7)
Vaginal parity	3 (2–4)
Diabetes mellitus	55 (17.2)
Urgency urinary incontinence	115 (35.9)
Prolapse reduction stress test	
Positive Negative	168 (52.5) 152 (47.5)
Midurethral sling ^a^	148 (46.3)
Type of prolapse surgery	
Colpocleisis Native tissue repair ^b^ Sacrocolpopexy	8 (2.5) 165 (51.6) 147 (45.9)

Values are presented as median (interquartile range) or number (%)

^a^ All procedures were performed on women with a positive stress test result in the manner of transobturator tape

^b^ Includes anterior colporrhaphy, iliococcygeus suspension, sacrospinous ligament fixation, and uterosacral ligament suspension

**Fig. 1:**
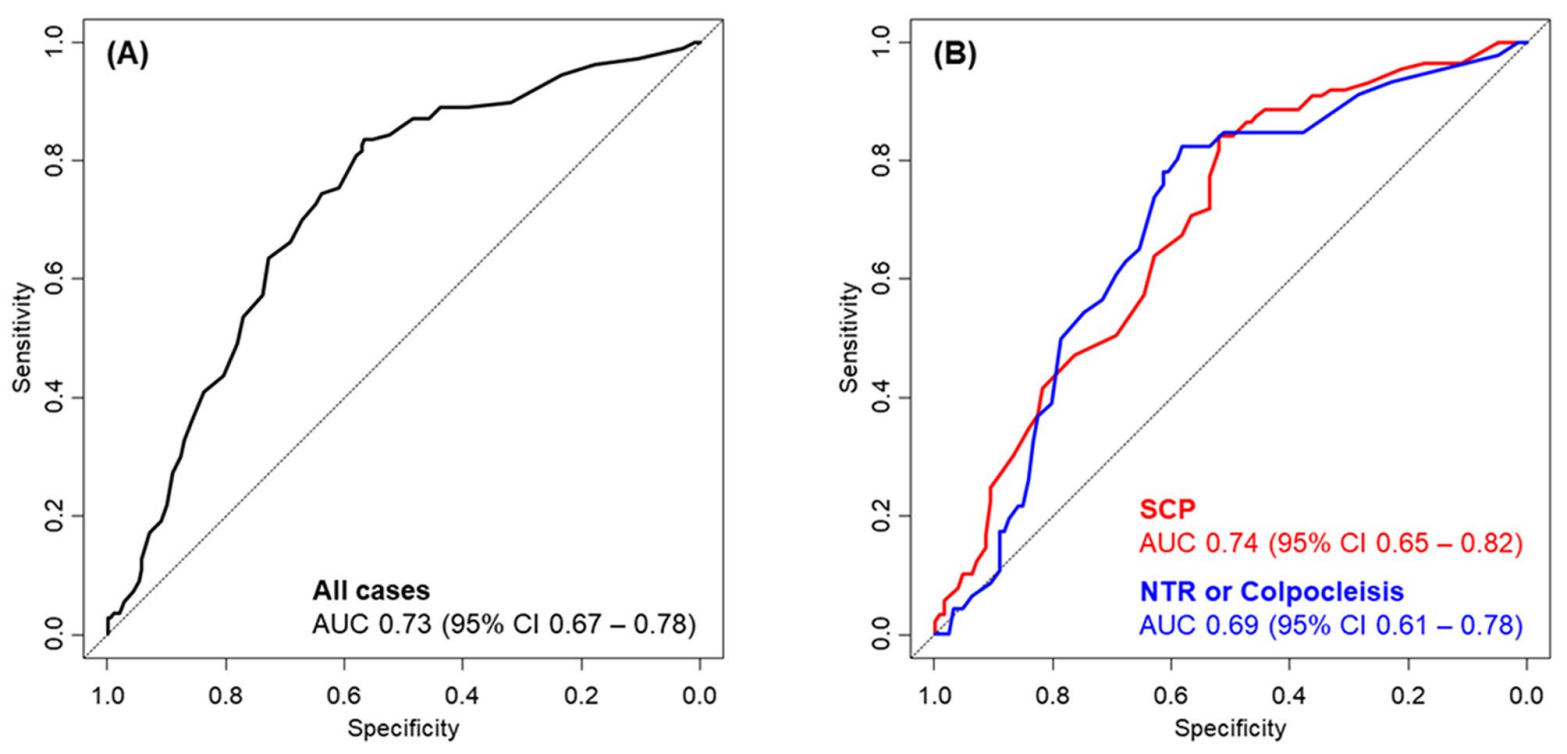
ROC curve of de novo SUI model. (**A**) Whole dataset. (**B**) According to the type of prolapse surgery. AUC: area under the curve; CI: confidence interval; NTR: native tissue repair; SCP: sacrocolpopexy

The overall incidence of de novo SUI was 34.4% (110/320): 26.6% (46/173) in women who underwent native tissue repair or colpocleisis and 43.5% (64/147) in those who had sacrocolpopexy; 12.8% (19/148) in women who received a MUS and 52.9% (91/172) in those who did not. The de novo SUI prediction model showed moderate discriminative ability in our study cohort; AUC (95% confidence interval [CI]) = 0.73 (0.67–0.78) in the whole cohort, 0.69 (0.61–0.78) in women who underwent native tissue repair or colpocleisis, and 0.74 (0.65–0.82) in those who had sacrocolpopexy (Fig. 1). There was no difference in the AUCs according to the type of prolapse surgery (DeLong’s test p = .48).

**Fig. 2:**
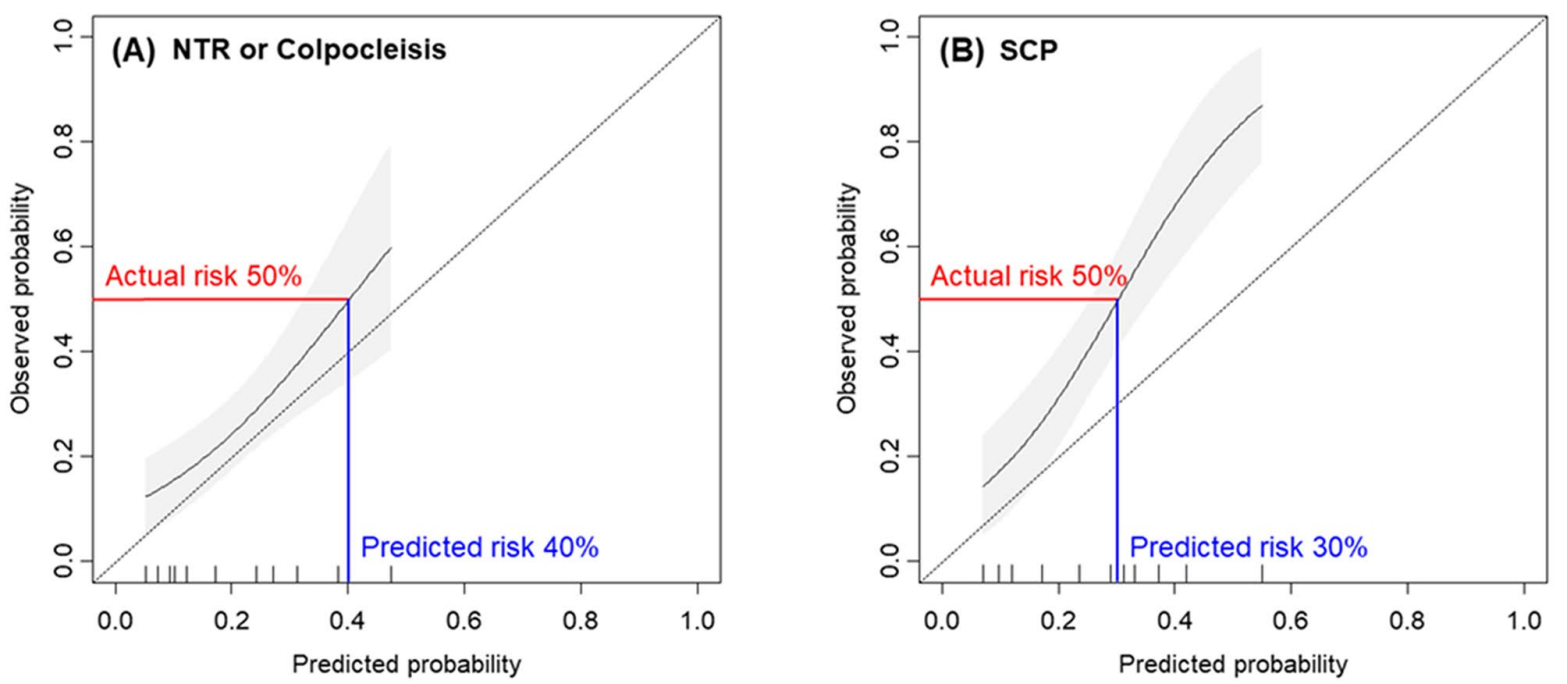
Calibration curve of de novo SUI model. (**A**) Native tissue repair or colpocleisis (**B**) Sacrocolpopexy. The grey shading indicates 95% confidence intervals

Calibration curves demonstrated that the model accurately predicted the observed outcomes of de novo SUI in women who underwent native tissue repair or colpocleisis but underestimated the actual risk in those who underwent sacrocolpopexy. The predicted probability cutoff points corresponding to an actual risk of 50% were 40% in women who underwent native tissue repair or colpocleisis and 30% in those who underwent sacrocolpopexy (Fig. 2).

## Discussion

This study externally validated the de novo SUI model in Korean women and showed moderate discrimination in either the whole study cohort or subgroups according to the type of prolapse surgery. The model was well calibrated in women who underwent native tissue repair or colpocleisis but underestimated the risk in those who underwent sacrocolpopexy.

A de novo SUI model was developed to provide individual risk estimates for the development of de novo bothersome SUI symptoms during the first year after prolapse surgery, thereby aiding in shared decision-making regarding prophylactic continence surgery. Two clinical trials conducted by the National Institutes of Child Health and Human Development (NICHD) Pelvic Floor Disorders Network were used for the development and validation of the model: OPUS (for the development and internal validation) and the Colpopexy and Urinary Reduction Efforts (CARE) (for the external validation) trials. The concordance index analogous to AUC was 0.73 (95% CI 0.65–0.80) and 0.62 (95% CI 0.56–0.69) in the OPUS and CARE trials, respectively [5].

Thereafter, three studies were conducted to validate the de novo SUI model and test its accuracy in an international cohort independent from the NICHD Pelvic Floor Disorders Network. One study evaluated the model’s performance on 152 Dutch patients who participated in the CUPIDO (Concomitant surgery and Urodynamic investigation in genital Prolapse and stress Incontinence. A Diagnostic study including Outcome evaluation) trials and revealed that the model’s discrimination was acceptable and consistent with performance in the original model development cohort [6]. The concordance index was 0.63 (95% CI: 0.52–0.74). The other two studies analyzed 169 Spanish and 225 Turkish patients who underwent vaginal prolapse surgery, and the concordance index was found to be 0.69 (95% CI: 0.58–0.80) and 0.56 (95% CI: 0.35–0.77), respectively [7, 8]. Different from the original model, they assessed the presence of SUI at 1 year as the primary outcome rather than the presence of SUI at any point within the 12 months. Bothersome SUI symptoms may spontaneously remit during the follow-up period. The rate of de novo SUI was low in the latter study (5.3%), and spontaneous remission of SUI symptoms might result in decreased discriminative ability of the model.

Consistent with the original model development cohort [5], the model showed good calibration in our study population that underwent native tissue repair or colpocleisis. However, we found that the model underestimated the actual risk in patients who underwent sacrocolpopexy. Limited bladder neck and urethral mobility due to mesh traction may diminish urethral compression during stress and contribute to an increased occurrence of de novo SUI [12, 13]. We further analyzed calibration curves to define a probability cutoff point reasonable to offer prophylactic continence surgery. The predicted probability cutoff points corresponding to an actual risk of 50% were 40% in women who underwent native tissue repair or colpocleisis and 30% in those who underwent sacrocolpopexy.

This is the first study to validate the prediction accuracy of the de novo SUI model in an Asian population. A large sample size with a higher prevalence of de novo SUI made our study more powerful and enabled conclusive validation of the model. Furthermore, we performed subgroup analyses by surgery type and could define probability cutoff points reasonable to offer prophylactic continence surgery. Nonetheless, there were some limitations, mainly attributable to the inherent weakness of a retrospective study. Concomitant midurethral sling was performed only on women with a positive preoperative prolapse reduction stress test result, which might affect our findings. However, given the low negative predictive value of the preoperative prolapse reduction stress test for postoperative SUI [4], our findings may be helpful in decision-making for prophylactic anti-incontinence surgery in women without occult SUI.

## Conclusion

The de novo SUI prediction model is acceptable for use in Korean women and may aid in shared decision-making regarding prophylactic continence procedure at the time of prolapse surgery.

## Data Availability

The datasets used and/or analyzed during the current study available from the corresponding author on reasonable request.

## Abbreviations

AUC: Area under the receiver operating characteristic curve
CARE: Colpopexy and Urinary Reduction Efforts
CI: Confidence interval
CUPIDO: Concomitant surgery and Urodynamic investigation in genital Prolapse and stress Incontinence. A Diagnostic study including Outcome evaluation
MUS: Midurethral sling
NICHD: National Institutes of Child Health and Human Development
OPUS: Outcomes Following Vaginal Prolapse Repair and Midurethral Sling
PFDI: Pelvic Floor Distress Inventory
POP: Pelvic organ prolapse
SUI: Stress urinary incontinence
